# Marketing evidence‐based surgery

**DOI:** 10.1111/ans.18006

**Published:** 2022-12-05

**Authors:** Joshua G. Kovoor, Aashray K. Gupta, Jacob Cherini, Stephen Bacchi, Guy J. Maddern

**Affiliations:** ^1^ Information in Surgery Journal Club Adelaide South Australia Australia

“Markets are Conversations”

Rick Levine, Christopher Locke, Doc Searls, and David Weinberger

*The Cluetrain Manifesto* (2009)^1^

Human attention is influenced by a combination of implicit priming by preceding knowledge and novel information.^2^ Marketing involves a set of processes for creating, communicating, and delivering value to customers, while also managing customer relationships to benefit the organization or entity and its stakeholders.^3^ Marketing can influence human behaviour as it affects not only sensory perception, but also social perception, which is the neural processing of data to analyse the dispositions and intentions of others.^4^ The volume of marketing in healthcare by healthcare professionals, associations and corporations has increased dramatically in recent decades, particularly the advertising of health services and pharmacological interventions delivered directly to consumers.^5^ As expected, in this time the utilization of medical services and products has increased. Digitalisation has allowed information to flow more freely and rapidly than ever before. This distribution of information has been accelerated by social media. Social media is used by billions of individuals worldwide. Through an environment in which vendors can directly interact with consumers social media provides one of the most powerful marketing tools in history.^6^ When marketing any concept or product on social media, the “social feedback cycle” is engaged, whereby a funnel of attention directly connects operations and marketing via customer‐driven digital social interactions.^7^ In the modern era, the rapid information flow through digital and social media environments can be used to widely disseminate evidence‐based concepts and initiate clinical innovation.

Evidence‐based surgery is the practice of using reliable scientific data to deliver surgical care for optimal patient and system outcomes and to advance surgical care at an individual, community, and population level. Global engagement with evidence‐based surgery has followed the rapid progress of the evidence‐based medicine paradigm,^8^ but entails unique challenges to implementation primarily due to the safety, efficacy and effectiveness considerations associated with surgical intervention.^9^ A significant increase in the international knowledge and utilization of evidence‐based surgical principles followed the publication of the innovation, development, exploration, assessment, and long‐term study (IDEAL) model of surgical innovation in 2009.^10^ The method of publication as a *Lancet* Series had a widespread impact and was read throughout surgical academia. The principles were developed by internationally‐renowned surgical experts within the Balliol Collaboration who have gone on to promote these within their respective local and national jurisdictions. The resultant engagement with these recommendations is reflected by the primary paper within the *Lancet* Series^10^ being cited over 1000 times (Scopus), and the recommendations themselves being refined on multiple occasions and published in internationally‐read peer‐reviewed journals.^11^, ^12^ In a sense, this ‘marketing’ of evidence‐based surgery has resulted in a global change in practice and immeasurable follow‐on benefits for patient and system outcomes.

To be effective, marketing needs to adapt methodologies to incorporate modern technologies. Electronic data and information technology are now firmly integrated within the foundation of modern healthcare systems.^13^ Digitalisation has ensured that within the academic surgical community, there is now greater access to peer‐reviewed scientific literature. Peer‐reviewed journals worldwide, including the *ANZ Journal of Surgery*, have reflected this, by reducing physically‐printed information and moving towards the dispersion of data via digital media alone.^14^ The dissemination of peer‐reviewed information via online services has been shown to increase access and availability of the contained data.^15^ Accordingly, the implementation of the conveyed evidence‐based surgical principles within clinical practice is also likely to increase. Recent years have seen most surgical institutions and groups worldwide form official online and social media presences for the purpose of promoting interpersonal engagement with their services. The formation of online communities using social medial can be effective in promoting evidence‐based surgical principles regardless of clinical seniority. At the junior level, medical student and junior doctor societies can use online forums and social media communities to facilitate the provision of information to those interested in future surgical careers. Models of providing education in evidence‐based surgery can also be delivered effectively with social media as a facilitative mechanism.^16^ At the senior level, surgical colleges can use websites and digital platforms to produce and communicate guidance statements at a local, national, and even international level. As with academic journals, scientific conferences and events can now reach a much larger audience internationally via broadcasting online.

The philosophy fundamental to evidence‐based surgery is adaptable to the digitalisation movement, and can even be employed to enhance the utilization of electronic data and information technology within surgical systems of care. To optimize the potential for information dispersion that accompanies the use of digital data, surgical groups and institutions should look to harness technology to market evidence‐based principles wherever possible. As seen with the success of the Balliol Collaboration in communicating a reliable structure for surgical innovation to the global surgical community,^10^, ^11^, ^12^ less‐experienced and less‐renowned groups and individuals can do their part and market these ideas to promote consumer engagement in their local jurisdictions.^17^ The utilization of online services and social media platforms to facilitate this dissemination of surgical knowledge is still in the early stages of adoption. However, the future potential for local and global evidence‐based surgery is substantial.

## Author contributions

**Joshua G. Kovoor:** Conceptualization; investigation; writing – original draft; writing – review and editing. **Jacob Cherini:** Conceptualization; investigation; writing – review and editing. **Stephen Bacchi:** Conceptualization; investigation; writing – review and editing. **Guy J. Maddern:** Conceptualization; investigation; supervision; writing – review and editing.
